# Development of fiber optic *in vitro* release testing method for dexamethasone release from the oil solutions

**DOI:** 10.5599/admet.1465

**Published:** 2022-10-11

**Authors:** Filip Kozlina, Ivan Meštrović, Viktor Novak, Nikola Marjanović, Biserka Cetina-Čižmek

**Affiliations:** 1 University of Zagreb, Faculty of Pharmacy and Biochemistry, A. Kovačića 1, 10 000 Zagreb, Croatia; 2 PLIVA Croatia Ltd, TEVA Group Member, Prilaz baruna Filipovića 25, 10 000 Zagreb, Croatia

**Keywords:** dissolution, prolonged release injection, parenterals, derivative spectroscopy

## Abstract

For many parenteral drugs, there is still no standardized method for in vitro release (IVR) testing available. This article presents the development of a new IVR method for oil solutions using a dialysis membrane and USP II apparatus coupled to a fiber optic UV-Vis spectrometer. Experiments were performed using dexamethasone formulations containing castor oil as a solvent with the addition of cosolvents, 20 % (v/v) of isopropanol or Capryol^®^ 90. Based on solubility testing results, castor oil was chosen as the best solvent amongst other vegetable oils, while a significant increase in solubility was obtained by adding either of the two cosolvents. Partitioning experiments were performed to ensure these formulations could achieve prolonged drug release. IVR testing was performed with model formulations and critical test parameters were varied in order to examine the method’s sensitivity. The developed method was sensitive to temperature and stirring rate, while coupling the USP II apparatus with a fiber optic UV-Vis spectrometer enabled complete automation. Moreover, due to the interference of excipients on fiber optic detection of dexamethasone during the release testing, derivative spectroscopy was successfully introduced for the elimination of the interference. The developed IVR method described herein could be useful in preformulation investigations and the early development of novel formulations.

## Introduction

The release of an active pharmaceutical ingredient (API) from a drug product is a prerequisite for all pharmacokinetic processes as well as the therapeutic effect of an administered drug. *In vitro* release (IVR) testing is often conducted early in the development of drug products as it can potentially give insight into the viability of the drug product and, preferably, predict its *in vivo* behaviour. Furthermore, IVR test plays an important role in quality control and regulatory processes for some drug products [1-4]. Several standardized methods of conducting IVR tests are described in The United States Pharmacopoeia (USP), European Pharmacopoeia (Ph. Eur.) and Japanese Pharmacopoeia (JP), but their application is mostly limited to orally administered drug products and transdermal patches. To date, no standardised IVR test for the parenteral products is available, which makes the development of such methods an enduring challenge [5,6].

Oil solutions are a type of prolonged-release dosage form that allows parenteral administration of poorly soluble drugs or unstable in aqueous systems [7]. Vegetable oils (such as castor, sesame, cottonseed, etc.), medium chain triglycerides and fatty acid monoesters, or their mixtures normally play the role of a solvent in these products [8]. Following their application (usually intramuscular), the oil forms a depot, from which a prolonged drug release (and some excipients) occurs. The prolonged drug release allows for a significantly less frequent application, making oil solutions especially suitable for the treatment of chronic diseases (such as hypogonadism), as well as diseases characterised by variable patient adherence (e.g. schizophrenia) [9,10]. When developing an oil solution, the key points to think of are the solubility of the drug in the lipophilic vehicle, its viscosity, and the partitioning of the drug between the oil and the aqueous phase. By increasing the solubility of the drug, a higher concentration of the drug in the solution is allowed, and thereby administration of a lower volume of the such drug product. This resolves some of the possible major issues regarding this type of formulation, including pain and irritation at the injection site. Solubility of the drug is most often increased using cosolvents, usually, alcohols or simple esters, whose addition simultaneously decreases the viscosity of the solution, making it easier to flow through a hypodermic needle (raising the syringeability of the solution). However, these cosolvents allow higher partitioning of the drug into the aqueous phase, which might shorten the release time of the drug, thus decreasing its value as a sustained release formulation. Therefore, the amount of cosolvent added to the solution must be carefully determined in order to balance out its positive effects on drug solubility and solution viscosity while maintaining the partitioning of the drug adequate for prolonged drug release [8].

Dexamethasone (DEX) is a highly potent glucocorticoid used in the treatment of multiple conditions characterised by severe inflammation, e.g., rheumatoid arthritis. DEX is a moderately lipophilic molecule with a log *P* of 1.68, practically insoluble in water and sparingly soluble in ethanol. Its most acidic group has a p*K*a value of 12.42, meaning it is a neutral molecule within the physiological pH range [11]. Oil solution-based drug products containing DEX have been investigated in several studies so far [12,13], as such setup might prove useful in the treatment of inflammatory diseases due to its potential prolonged release. Furthermore, such a solution, applied locally, should pose less of a risk due to drug systemic exposure. Considering the fact that DEX contains a chromophore, its content can be determined via UV-Vis spectrometry.

Analysis on the standard USP II dissolution system requires sampling the medium at desired time points, followed by filtration and further analysis. Sample analysis can be a significant drawback in time and resources if performed by HPLC analysis. On the other hand, an online UV-Vis spectrometer coupled with a dissolution system significantly decreases the time needed for obtaining drug release profile curves. The use of a fiber optic automated dissolution system has many advantages compared to both of these options.

Fiber optic probes connected to a UV-Vis spectrometer are immersed into the release medium throughout the entire experiment, thus not requiring additional medium sampling at desired time points and concurrent replacement of the fresh medium. This results in significantly lower consumption of time and materials, such as filters, pipettes, or tubing, compared even to an online spectrometer detection, and especially to manual sampling followed by HPLC analysis. Simplified detection also decreases the intervals between time points, making it possible to obtain readouts even in less than a minute intervals if desired. Finally, in-situ spectroscopic detection through optic probes enables real-time monitoring of drug release during the dissolution process [14].

Even though the probe is constantly exposed to room light (unlike conventional cuvettes), fiber optic spectrometry has so far proven to be mostly unaffected by this potential interference. Due to the lack of release medium filtration, detection can be compromised by the interference of unsolved particles or excipients, which can cause light scattering on the optic probes [14]. These interferences can be resolved with the use of mathematical filters, such as baseline correction or derivative spectroscopy [15], as demonstrated in this article. In addition, with the use of an appropriate dialysis bag as a sample container, possible interferences in detection can be minimized or totally diminished.

Some previous attempts at developing an IVR method for oil solutions utilised a dialysis cell model [16,17] and a simple magnetic stirrer setup with the oil formulation dispersed in a release medium [18]. In general, the IVR methods for oil solutions can be divided into (a) those with the oil solution floating atop the release medium, (b) dialysis methods and (c) continuous flow methods. All these approaches share some of the same possible issues. Oil solution tends to float atop the release medium and stick to parts of the apparatus. This problem is completely avoided using dialysis bags, and furthermore, the oil packed in a dialysis bag imitates the depot that is formed *in vivo* after administration in muscle tissue [8].

This article describes the development of the IVR method using a dialysis bag placed in a paddle apparatus (commonly known as the USP II apparatus) coupled to a fiber optic spectrometer. The aim of such a setup is to provide an automated IVR method utilising a widely available apparatus, avoiding potential sampling and sink condition-related problems by design, for the screening of various formulations in the early development phase.

## Experimental

Refined oils used in the solubility and IVR experiments were purchased from Croda (United Kingdom) and include corn oil, peanut oil, cottonseed oil, sesame oil and castor oil. DEX powder was purchased from Pfizer (USA). Cosolvents propylene glycol monocaprylate type II (Capryol® 90) and isopropanol were purchased from Gattefosse (France) and Merck (Germany), respectively. Tetrahydrofuran and acetonitrile were obtained from Merck (Germany) and used for sample preparation for HPLC analysis. Regenerated cellulose (RC, MWCO (molecular weight cut-off) 50 kDa) and cellulose ester (CE, MWCO 300 kDa) dialysis membranes, used as formulation carriers, were obtained from Spectrum Labs (USA). Regenerated cellulose filter membranes used for medium degassing were obtained from GE Healthcare (USA). Demineralized water was used throughout the study. All chemicals and solvents were of analytical grade. For the determination of partition coefficient and medium in IVR studies, phosphate buffer pH 7.4, made from sodium hydroxide and potassium dihydrogen phosphate, obtained from Kemika (Croatia), was used. Ethanol was obtained from Kemika (Croatia).

### Solubility of DEX

Solubility of DEX was determined in five different vegetable oils (castor, peanut, cottonseed, sesame and corn), pure or mixed with 10 and 20 % (v/v) of one of the cosolvents (Capryol® 90 or isopropanol) by dissolving an excess amount of DEX in 3 mL of the solvent/mixture using a shaker at 37 °C and 100 rpm for 24 hours. 1 mL of each supernatant was sampled after 2 hours and 24 hours and then centrifuged at 10000 rpm for 15 minutes to remove the undissolved DEX. After centrifugation, 200 μL of supernatant was diluted with 600 μL of tetrahydrofuran and 800 μL of acetonitrile in vials for the HPLC analysis. All samples were prepared in triplicates. Castor oil combined with 20 % (v/v) of isopropanol and castor oil with 20 % (v/v) of Capryol® 90 were chosen as the best formulations for the following experiments.

### Determination of partition coefficients

*P*_app_ (apparent partition coefficient) was used to quantify the partitioning of DEX in test solutions. Equation (1) describes the calculation of Papp:


(1)

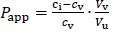



where c_i_ represents the concentration of prepared oil solution for determination of *P*_app_, *c*_v_ represents the concentration of drug in the aqueous phase (phosphate buffer pH 7.4) at equilibrium, while *V*_v_ and *V*_u_ are volumes of aqueous and oil phase at the end of the experiment, respectively [8].

The partition coefficient between the formulation (castor oil, cosolvent and DEX, representing the oil phase) and the phosphate buffer pH 7.4 (representing the aqueous phase) was determined by mixing them at 1:1 volume ratio and shaking at 37 °C and 100 rpm for 24 hours. The concentration of added DEX was determined previously in the solubility study (1 mg/mL in Capryol® 90 mixture and 2 mg/mL in isopropanol mixture). After 2 and 24 hours, oil and aqueous phases were sampled (1 mL) and centrifuged for 15 minutes at 10000 rpm. The obtained supernatant was diluted as already described in the solubility experiments. All samples were prepared in triplicates.

### HPLC analysis

Samples from the solubility and partitioning studies were analysed on HPLC system Agilent 1100/1200 series connected to diode array detector adjusted to 241 nm. Kinetex C18 column (4.6 x 50 mm, particles 2.6 μm and pore size 100 Å) by Phenomenex (USA), heated to 40 °C, was used. The chromatograms were obtained with a gradient elution, where acetonitrile and buffer pH 2.0 were components of the mobile phase. Injector temperature was adjusted at 35 °C, the flow rate at 1.8 ml/min and sample injection volume at 10 μL. The stock solution used in solubility and partitioning studies was prepared by dissolving 15 mg of DEX in 25 mL of tetrahydrofuran. The two working standard solutions were obtained by further diluting the stock solution. The first one was obtained by diluting 5 mL of stock solution in 25 mL of acetonitrile and was used for assay of DEX in castor oil solutions. The second standard solution was prepared by diluting 1 mL of stock solution in 100 mL acetonitrile. It was used for DEX assay in solutions composed of four other oils.

### In vitro release

IVR experiments were performed using a USP II apparatus coupled to the fiber optic UV-Vis spectrophotometer (Agilent, Germany) for real-time absorbance measurement. The 20 mm optical path probes were used for precise online absorbance determination. Single point detection at 241 nm was used for the calculation of released DEX, while a spectrum scan from 200 to 300 nm at each time point was used later on for derivative spectroscopy. Phosphate buffer pH 7.4 was used as a release medium, degassed before every experiment following the USP procedure [19], since the non-degassed medium showed unexpected results due to light scattering caused by bubbles of entrapped air in the fiber optic probes.

Before each experiment, dialysis membranes were soaked in the dissolution medium for thirty minutes. The membranes were cut in sizes compatible with the intended amount of test solutions. Prior to every experiment, for precise absorbance determination, the spectrophotometer baseline was determined by measuring the dissolution medium absorbance. Subsequently, the absorbance of prepared standard solutions was also determined for each of the six used probes individually to adequately calibrate the optic fibres.

Standard solutions were prepared by dissolving 10 mg of DEX in 25 mL of tetrahydrofuran and further diluted with the dissolution medium to 100 mL or 200 mL final volume, resulting in 0.004 mg/mL or 0.002 mg/mL final concentration of DEX in the standard solutions for isopropanol or Capryol® 90 test solutions, respectively. This was equal to the concentration of DEX in the release medium in the case of 100 % DEX release.

Samples for IVR analysis were prepared by dissolving DEX in each of the two oil formulations to the concentrations obtained within the solubility study. Concentration of DEX in castor oil containing 20 % (v/v) isopropanol was 2 mg/mL, while in the castor oil containing 20 % (v/v) Capryol® 90, it was 1 mg/mL. Samples were tested in each experiment in triplicates at least.

Dialysis bags were sealed at one end with a weighted pin and 1 mL of the formulation was added. They were later carefully pinned on the other side without trapping air bubbles inside the bags. Absorbance measurements were performed at 5, 10, 15, 20, 30, 45, 60, 90, 120, 150, 180, 240, 300, 360, 720 and 1440 minutes to determine the drug dissolution profile at given parameters during the method development. For further studies, additional sampling was performed every 12 hours up to a maximum of 72 hours. As a part of the development of the optimal dissolution method, sensitivity to several parameters was tested: type of dialysis membrane, temperature, release medium, stirring rate, the composition of the release medium, and test solution volume.

## Results and Discussion

### Solubility

**Figure 1.**
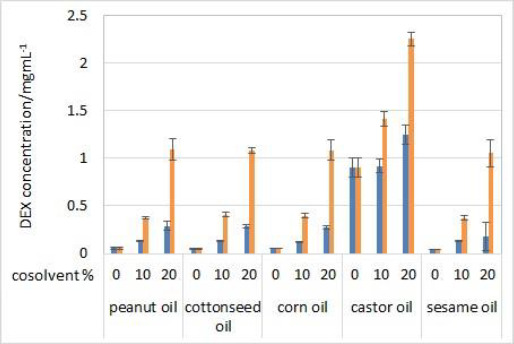
Solubility of DEX after 24 hours incubation in various vegetable oils alone and with the addition of cosolvents (Capryol® 90 (blue bars) or isopropanol (orange bars) at 10 % or 20 % (v/v)). Error bars represent standard deviation, n=3.

Solubility of DEX was determined in five oils (solvents) and mixtures with 10 or 20 % (v/v) of one of the cosolvents (isopropanol or Capryol® 90). Evaluated oils are commonly used as carriers for parenteral oil depots, while selected cosolvents serve as model type of excipients that can increase the solubility of the drug in selected oil in order to achieve model drug therapeutic concentrations and obtain satisfactory partitioning for prolonged release. No significant differences in solubility were observed between 2 and 24 hours of incubation, indicating that the equilibrium occurs quickly after the addition of DEX to the solvent mixtures. Therefore, only the results for solubility after 24 hours are shown (Figure 1).

DEX did not show high solubility in pure vegetable oils, except for castor oil. The composition of triglycerides in corn, cottonseed, sesame and peanut oil is very similar and the fatty acid moieties mostly come from oleic and linoleic acid. Castor oil is much more hydrophilic due to the high content (about 90 %) of ricinoleic acid residues amongst the triglycerides. Ricinoleic acid contains one hydroxyl group, which enables hydrogen bonding with DEX's H-bond acceptor groups [20].

Cosolvent addition clearly made a significant impact on the solubility of DEX. Solubility greatly increased with the addition of 10 % (v/v) of both cosolvents and increased with the 20 % (v/v) concentrations. DEX was more soluble in isopropanol than Capryol® 90 solutions, which was expected due to the hydrophilic nature of isopropanol. The highest solubility of DEX was achieved in castor oil solution with 20 % (v/v) of isopropanol (around 2.3 mg/mL) and 20 % (v/v) Capryol® 90 (around 1.2 mg/mL).

Based on the results of the solubility study, test solutions with castor oil as a solvent with the addition of 20 % (v/v) of one of the cosolvents were used for partitioning and IVR experiments.

### Partitioning

Determination of the partition coefficient is very helpful for the prediction of the behaviour of prolonged-release formulations *in vivo*. Partition coefficients determined for the chosen test solutions with the phosphate buffer pH 7.4 (later used as a release medium) as the aqueous phase are shown in Table 1. Log *P* value higher than 1 was obtained (1.25 for the isopropanol formulation and 1.53 for Capryol® 90 formulation), indicating that the formulations could achieve prolonged release. Furthermore, it took 24 hours of stirring for the isopropanol test solution to achieve equilibrium, while the Capryol® 90 test solution achieved equilibrium much earlier.

The appropriate choice of the type and concentration of cosolvent used in prolonged release parenteral product is reflected in both solubility and partition coefficient of the drug. Since better solubility and partitioning of DEX were observed using the formulation with 20 % (v/v) cosolvent, such test solutions were used for further IVR experiments. Higher concentrations of cosolvents might lead to a significantly lower partition coefficient, which could, in turn, diminish the prolonged release properties of the formulation.

### In vitro release method development

Following preliminary tests, several test parameters were varied in further method development in order to determine the method’s sensitivity and optimal test conditions.

### Dialysis membranes

**Figure 2.**
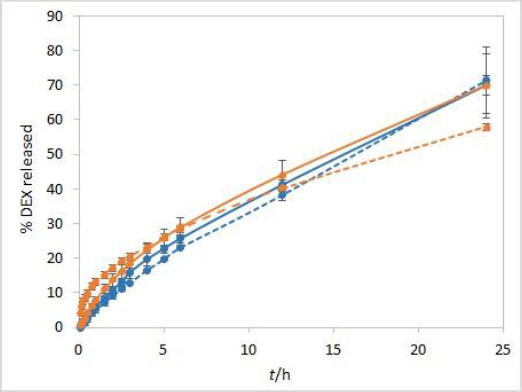
IVR profiles using two different types of dialysis membranes (CE with MWCO 300 kDa (solid line) and RC with MWCO 50 kDa (dashed line)) for isopropanol (orange) and Capryol® 90 (blue) test solutions. Other test parameters: 75 rpm, 37 °C, 500 mL of phosphate buffer pH 7.4 release medium. Error bars represent standard deviation, n=3.

During the method development, two types of dialysis membranes were inspected, CE membranes with MWCO 300 kDa and RC membranes with MWCO 50 kDa. When comparing dexamethasone release profiles from isopropanol and Capryol® 90 containing formulations, the great similarity was observed when CE membranes were used, while a certain difference between the profiles was noticeable with RC membranes which have a smaller pore size and contact area (Figure 2). Since the concentration of DEX is twofold higher in isopropanol compared to Capryol® 90 containing formulation, the concentration gradient is consequently higher, which results in faster DEX release from the isopropanol-containing formulation in the case where RC membranes are a rate-limiting factor.

### Temperature

**Figure 3.**
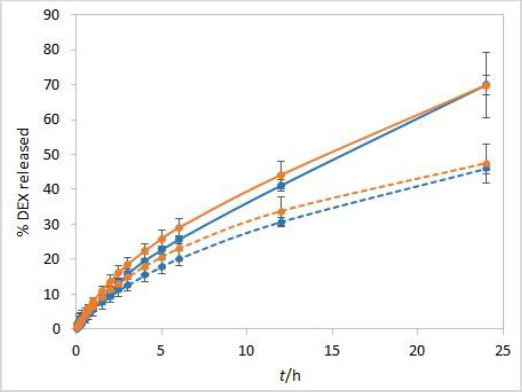
IVR profiles at two different temperatures (25 °C (dashed line) and 37 °C (solid line)) for isopropanol (orange) and Capryol® 90 (blue) test solutions. Other test parameters: CE membranes, 75 rpm, 500 mL of phosphate buffer pH 7.4 release medium. Error bars represent standard deviation, n=3.

The sensitivity of the method to temperature conditions was examined using both test solutions at 25 °C and 37 °C. Following basic physical principles, a slower release is expected at a lower temperature due to slower rates of every process involved in DEX release into the medium. IVR profiles presented in Figure 3 confirm this hypothesis and show that the method is sensitive to temperature. Controlled temperature during the test is a critical parameter for this method.

### Release medium stirring rate

**Figure 4.**
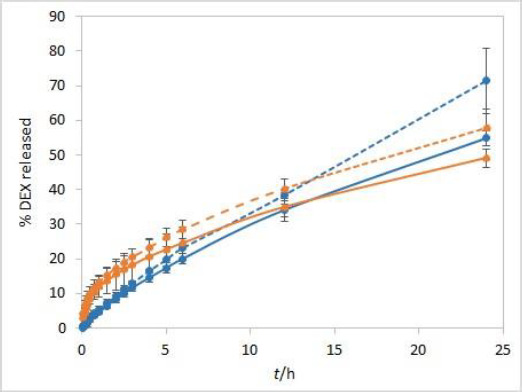
IVR profiles at two different stirring rates (50 rpm (solid line) and 75 rpm (dashed line)) for isopropanol (orange) and Capryol® 90 (blue) test solutions. Other test parameters: RC membranes, 37 °C, 500 mL of phosphate buffer pH 7.4 release medium. Error bars represent standard deviation, n=3.

The stirring rate is considered an essential parameter for IVR methods and must be carefully chosen and evaluated. Unexpected results, which might arise using an insufficient stirring rate, include thickening of the diffusion layer, leading to a lowered concentration gradient and diffusion potential and finally resulting in a lower release rate. On the other hand, if the stirring rate is too high, the method loses its discriminatory power. Using the USP II apparatus, the FDA recommends a stirring rate of 50 to 75 rpm [1]. Therefore, the release was tested using these two stirring rates. The method is not very sensitive to different stirring rates. However, slightly higher drug release rates were observed when higher stirring rates were applied (Figure 4).

### Composition of the release medium

Aiming to reduce the method duration, DEX release from test solutions was analysed using a release medium containing 10 % (v/v) ethanol in phosphate buffer pH 7.4. Sink conditions were ensured during these analyses as well. The solubility of DEX in phosphate buffer pH 7.4 with 10 % (v/v) ethanol is 0.17 mg/mL [21]. According to the USP criteria, the volume of the release medium to ensure sink conditions for DEX should be 238 mL, while to Ph. Eur. criteria in the range from 238 mL to 473 mL.

**Figure 5.**
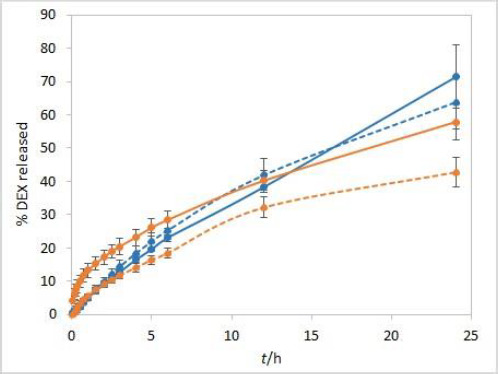
IVR profiles using phosphate buffer (pH 7.4) medium alone (solid line) and modified medium with 10 % (v/v) ethanol (dashed line) for isopropanol (orange) and Capryol® 90 (blue) test solutions. Other test parameters: RC membranes, 37 °C, 75 rpm, 500 mL of release medium. Error bars represent standard deviation, n=3.

Considering that DEX is more soluble in ethanol than in water solutions, it was presumed that the release rate is going to be higher in the medium with the addition of ethanol than in the phosphate buffer alone. However, the results were entirely different (Figure 5). The overall percentage of DEX released after 24 hours was lower for both test solutions using the release medium with 10 % ethanol and even throughout the entire test for the isopropanol test solution. This is probably due to the fact that ethanol itself can diffuse from the release medium into the test solution through the dialysis membrane pores, thus increasing the solubility of both DEX and cosolvent and lowering DEX partitioning into the release medium, as well as its release rate. This effect is more pronounced with the isopropanol compared to the Capryol® 90 test solution, presumably due to its alcoholic nature.

### Volume of test solution

**Figure 6.**
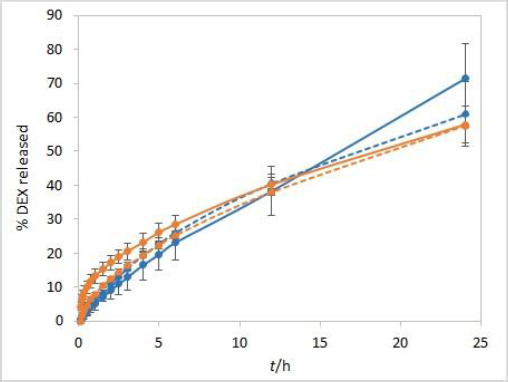
IVR profiles using 1.0 mL (solid line) and 0.5 mL (dashed line) of test solutions for isopropanol (orange) and Capryol® 90 (blue) test solutions. Other test parameters: RC membranes, 37 °C, 75 rpm, 500 mL of phosphate buffer pH 7.4 release medium. Error bars represent standard deviation, n=3.

Development and evaluation of IVR methods often include evaluation of the sample volume on release, defining whether the method is sensitive to this parameter. For this purpose, a twice lower volume of the test solution (500 μL) when compared to other experiments was also analysed. When using a smaller sample volume of test solutions with RC membranes, great similarity between the IVR profiles of both tested formulations was observed (Figure 6). In these conditions, RC membranes are no longer a rate-limiting factor. On the other hand, CE membranes with larger pore sizes are not a rate-limiting factor even when 1 mL of test solution is used (Figure 2).

### Complete IVR profiles using the optimized test parameters

**Figure 7.**
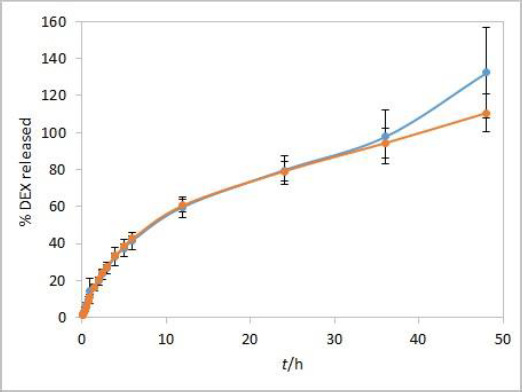
Complete DEX IVR profiles for Capryol® 90 (blue) and isopropanol (orange) containing test solutions in castor oil. Test parameters: CE membranes, 37 °C, 75 rpm, 1 mL sample volume, 500 mL of phosphate buffer pH 7.4 release medium. Error bars represent standard deviation, n=6.

Method development results indicate that the method is sensitive to temperature and moderately to stirring rate but not to other evaluated parameters. Additional analyses were performed with both test solutions to determine the method reproducibility and overall length of the test required to observe the complete release of the drug. Since the main purpose for using dialysis membrane in our method was avoiding interference from formulation excipients and mimicking of oil depot that forms after administration *in vivo*, we didn’t want the choice of dialysis membrane type to be a rate-limiting factor. Based on the results observed between the profiles when comparing the two types of membranes, and due to their easier handling, CE membranes were chosen as a better option for the IVR testing method. The test parameters used were as follows: CE membranes, 37 °C (physiological temperature, higher release rate compared to 25 °C), phosphate buffer (pH 7.4) as release medium (biorelevant regarding pH, ensuring faster release compared to ethanol-containing medium), and 75 rpm stirring rate (ensuring moderately higher release rates compared to 50 rpm). 1 mL of each test solution was added to a dialysis bag. DEX release up to 48 hours for both test solutions is shown in Figure 7.

During the first few hours of testing, DEX is released at a higher rate than in the later ones. In the period between 6 and 12 hours, a change in the curve slope can be observed, indicating a point at which DEX release becomes nearly linear. These two phases in the IVR profiles are a consequence of the physicochemical properties of the cosolvent, which is well soluble both in the oil and the aqueous phase (in the case of isopropanol, the solubility of the cosolvent is even higher in the release medium). This leads to a release of the cosolvent into the release medium (alongside the drug) with kinetics different from those observed for the release of DEX alone.

Since the solubility of isopropanol in the release medium is significantly higher than the solubility of DEX, its release is faster than the release of DEX in the first hours, during which virtually all isopropanol becomes released to the medium. Consequently, the partitioning coefficient changes over time. When the rate of isopropanol release becomes constant or all isopropanol is released, DEX's solubility and partitioning coefficient become constant, leading to a linear terminal phase of DEX release. Furthermore, this linearity is maintained by a constant concentration gradient, which is guaranteed by design in this method, where sink conditions are ensured throughout the experiment.

Release kinetics from the oil phase to the oil/water interface in this type of delivery system is affected by drug diffusivity in the oil as well as convection induced by stirring in the aqueous phase [22]. IVR setup does have an influence on the convection process. However, the literature predominantly reports first-order release for oil solutions [22,23], which is also valid for profiles obtained in this study.

Two phases of release from oil formulations with a hydrophilic cosolvent were also observed *in vivo* in human blood, using a solution of nandrolone decanoate in sesame oil with benzyl alcohol as a cosolvent [24]. Reported biphasic IVR profiles nicely fit observed *in vivo* PK profiles.

Overall reproducibility (n = 6, from two different experiments) was achieved, quite acceptable for IVR method for modified release products (standard deviation below 10 % up to 48 or 24 hours for isopropanol and Capryol® 90 test solution, respectively). In addition, DEX release profiles from both test solutions were quite similar up to 36 hours, indicating that the type of cosolvent does not affect the release rate in these conditions.

On the other hand, the obtained DEX release profiles (Figure 7) were elevated and did not show a characteristic plateau pattern around 100 % at the final time points. This was even more pronounced at time points up to 72 hours, especially for Capryol® 90 test solution (data not shown). Since that was an indicator of interference during the absorbance reading with optic probes, this effect was further studied using the isopropanol test solution.

### Placebo interference

**Figure 8.**
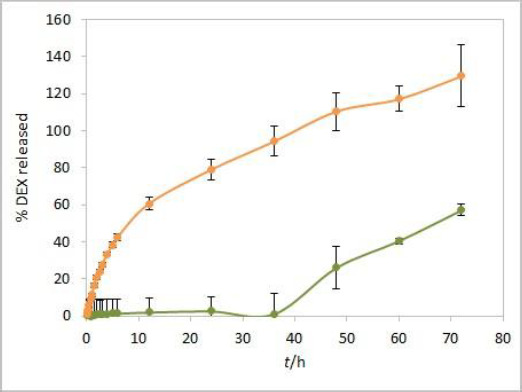
DEX release profile from isopropanol test solution (orange) compared to absorbance profile of respective placebo solution (green). Error bars represent standard deviation, n=3 (for placebo) and n=6 (for test solution).

An additional dissolution experiment was performed with a placebo solution containing only castor oil and isopropanol. The obtained absorbance profile up to 72 hours, corresponding to the percent of released DEX, was compared to the DEX release profile from the test solution (Figure 8). Increased absorbance of placebo was observed from 48-hour time point, indicating a significant effect of excipients from formulation on total absorbance readout in consecutive time points. The interference from the placebo is probably caused by components of castor oil that diffuse through the dialysis membrane at later time points. The absorbance intensity is more pronounced in placebo alone compared to the extent of interference in the test solution. That could be attributed to the slower diffusion of excipients from the test solution due to competition with faster diffusing DEX. Since the dialysis membrane of choice for performing the experiments was the CE membrane with MWCO 300 kDa, these interferences could probably have been reduced to a certain extent by using the RC membrane with MWCO 50 kDa. Instead, the elimination of interference by use of derivative spectroscopy is described in the following lines.

### Derivative spectroscopy

**Figure 9.**
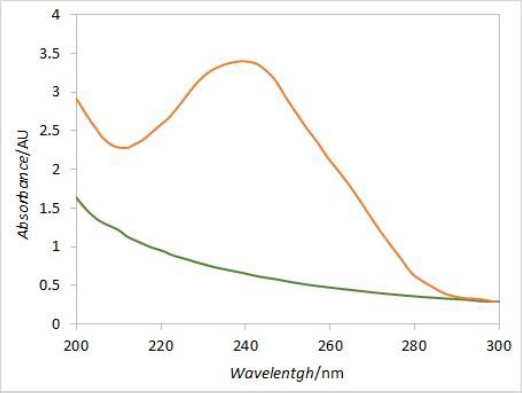
Comparison of DEX (orange) and placebo (green) UV spectra.

In order to eliminate the interference caused by excipient's absorbance at 241 nm, derivative spectroscopy was introduced. DEX UV spectrum is characterized by a local maximum of absorbance around 240 nm, while the placebo spectrum is predominantly flat around that wavelength (Figure 9). The first derivative curve of the DEX spectrum should therefore have two extremes at its peak inflection points, while the placebo would be predominantly flat with values around zero. Comparing the first derivative values (at the wavelength corresponding to the curve’s extreme) of standard solution spectra (i.e. 100 % dissolved DEX) with respective first derivative values of samples spectra at each time point, the DEX release curve without placebo interference should be obtained.

**Figure 10.**
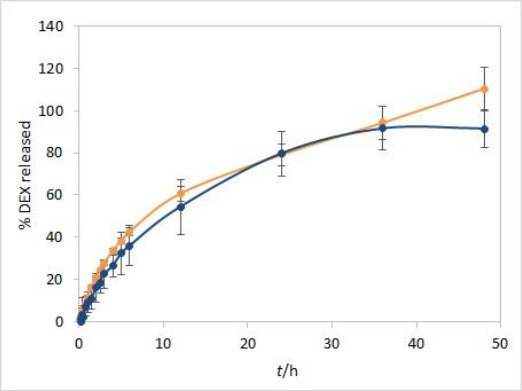
DEX average release profiles obtained by fiber optic detection at 241 nm (orange) and corrected to excipients interference by applying the first derivative of obtained spectra (dark blue). Error bars represent standard deviation, n=5.

In line with that, an additional IVR experiment was performed with the isopropanol test solution, according to the already described procedure. In addition to single point readout at 241 nm, spectrum scans from 200 to 300 nm were obtained for standard solution and samples at each time point in 5 vessels during the 48 hours of experiment duration. By applying the first derivative on spectrum scans, 252 nm was identified as the extreme value for obtained curves. The samples to standards ratio of first derivative values at 252 nm resulted in the DEX release profile corrected to excipients interference. The plateau with over 90 % released DEX was obtained at two finishing time points. When comparing the corrected average release profile to the one obtained by fiber optic detection, reasonable matching was obtained up to 36-hour time point (Figure 10).

Although the second derivative may be more suitable for extracting drug-specific information, in this case, more reliable results were obtained with the first derivative. Since dexamethasone spectrum has a local maximum around 240 nm, while placebo spectrum is predominantly flat in a wide range around that wavelength, the spectra first derivative resulted with local extremes at inflection points for dexamethasone while for placebo it was flat with values around zero. The local extreme at 252 nm was chosen for calculating dexamethasone release percentages by comparing the first derivative values of samples in each vessel with the respective standard first derivative values. Although a relatively narrow range window was obtained for the absolute values of first derivative curves at 252 nm, the resulting release curves were similar when comparing different vessels and reasonable matching was obtained between the average release curve and the one with no correction. Moreover, the interference from excipients at later time points was eliminated with the first derivative.

The second derivative curves were also calculated by using the same data. Since the window range is narrowed with every consecutive derivation step, the second derivative resulted in a range that was too narrow for reliable calculation. Therefore, the results obtained from the first derivative curves were used for interference elimination.

## Conclusions

The lack of a standardized IVR method for almost all types of parenteral drug products, including oil parenteral solutions, motivated us to develop one such method using a routinely used USP II apparatus coupled to a fiber optic UV-Vis spectrometer. DEX was used as a model drug. Test solutions containing castor oil as a solvent with the addition of 20 % (v/v) isopropanol or Capryol® 90 as a cosolvent were selected based on the results of solubility testing with several vegetable oils, as well as a partitioning study.

The solubility of DEX was much higher in castor oil than in other tested vegetable oils. Both isopropanol and Capryol® 90 proved to be efficient cosolvents, helping to achieve higher concentrations of DEX in test solutions (2 mg/ml and 1 mg/ml for isopropanol and Capryol® 90, respectively). Partitioning of DEX in these test solutions was adequate to ensure the prolonged release of DEX, and therefore they were chosen as model formulations.

The developed dialysis method has proven to be sensitive to temperature and moderately to stirring rate and has satisfying reproducibility with respect to USP standards for IVR methods. Furthermore, an automated system enables online detection and monitoring of the release through the fiber optic probes immersed in the dissolution medium for the whole duration of the experiment without the need for additional sampling and analysis. Packing the samples in dialysis bags helped avoid floating and sticking the oil solution to the apparatus, minimized the chance of interference with optic probe reading and acted as a “sample holder” mimicking *in vivo* distribution of the oil depot. However, most probably due to diffusion of excipients through the membrane, significant interference in reading was observed in later time points, more pronounced with a formulation containing Capryol® 90. Excipients interference was successfully eliminated by introducing derivative spectroscopy.

Both test solutions showed similar, prolonged release profiles. Taking into account that the concentration of DEX achieved in the solution containing isopropanol is twice the one achieved with Capryol® 90, it was concluded that the use of both cosolvents can lead to the development of potential prolonged release oil formulation in the form of solution. The choice of the type and amount of cosolvent determines the potential strength of the developed drug product.

The developed method described in this article may be used in oil-based drug product development for fast and direct early preformulation and formulation screening. Moreover, it serves as a basis for further research, leading to a standardised IVR method with significance in quality control and regulatory affairs purposes.

## Figures and Tables

**Table 1.** Results of partitioning study for DEX test solutions (SD – standard deviation, n=3).

Cosolvent	*t*/h	DEX conc. ratio (oil /aqueous phase)		log P
		Mean value	SD	
Isopropanol	2	21.1	2.1	1.25
	24	10.7	0.1	
Capryol^®^ 90	2	38.9	0.2	1.53
	24	34.7	1.8	
